# Understanding Psychosocial Wellbeing in the Context of Complex and Multidimensional Problems

**DOI:** 10.3390/ijerph17165937

**Published:** 2020-08-15

**Authors:** Francisco José Eiroa-Orosa

**Affiliations:** 1 Section of Personality, Assessment and Psychological Treatment, Department of Clinical Psychology and Psychobiology, Faculty of Psychology, University of Barcelona, Barcelona, 08035 Catalonia, Spain; fjeiroa@gmail.com or feiroa@ub.edu; 2 Yale Program for Recovery and Community Health, Department of Psychiatry, Yale School of Medicine, Yale University, New Haven, CT 06513, USA

**Keywords:** health systems, mental health, psychosocial well-being, sociocultural change, care systems transformation

## Abstract

This Special Issue deals with the topic of how people and social groups face problems in an increasingly complex and globalized society. The topics included in the call for papers were the interaction of psychosocial well-being and mental health with economic, gender, racial and ethnic inequalities, migration and demographic change and conflict and war, as well as the effects of stigma on people discriminated against because of their differential characteristics, whether they are of a sexual, disability or other minority. We made this proposal because we believed that, despite the introduction of the biopsychosocial model in the late 1970s as a paradigm of the integration of different disciplinary views, research in mental health and psychosocial well-being is still highly fragmented. For decades, we have tried to advance by emphasizing a part of the equation, with results that are at least modest. Therefore, in this Special Issue, we prioritized works aiming at disciplinary and methodological integration. The Special Issue was open to any subject area related to the impacts of social issues on mental health and psychosocial well-being. We were interested in empirical and theoretical enquiries at all ecological levels, from the psychosocial impact of social dynamics on individuals, to the analysis of how sociocultural and geopolitical factors influence health and collective psychosocial well-being.

## 1. Introduction

Psychosocial well-being is a superordinate construct that includes emotional or psychological well-being, as well as social and collective well-being [1,2]. The term “quality of life” is similar to psychosocial well-being in that it involves emotional, social and physical components. At the same time, it is often used in healthcare research to specify how the individual’s well-being may be impacted over time by a medical condition [3], thus muddying its conceptual clarity and specificity.

There have been various conceptual proposals [4], and even new designs of psychological interventions [5] that put psychological well-being in the center of mental health. Likewise, positive psychology, an approach based on enhancing happiness by focusing on the positive [6] and flourishing [7], could be said to represent a paradigmatic shift away from the deficit models that began two decades ago.

As positive as these steps may be, there have been methodological, conceptual, and even philosophical critiques raised by influential scholars [8] about these new approaches. Relatedly, it appears clear that the “positive” movement is predicated on a Eurocentric or Western point of view, although, recently, positive psychology has been incorporated into the cross-cultural research agenda [9]. Furthermore, there are authors that consider “positive” approaches [10] and, in general, Western psychological practice [11] and mental health systems [12] to be predicated on a context-free epistemology while simultaneously serving to reproduce the very structures and meanings that cause ill-being.

### The Current Special Issue

This Special Issue was proposed under the premise that there is a certain decontextualization and a lack of disciplinary integration in the measure of psychosocial well-being. That is, some of the research carried out in this field is developed looking for predictors or relationships between variables without fully considering the context in which these relationships occur or the possible biases that researchers or practitioners may have [13]. As we will see, the articles received consider this premise in different ways and with different intensities, but we are satisfied with the final outlook of the Special Issue. We have managed to compile a group of interesting studies on the measurement of well-being and/or its possible determinants in different contexts.

Although we do not include any articles on the subject, since the closing of the reception of articles was set at the end of 2019, the best example of the need to understand psychosocial well-being in a changing and complex context has been the COVID-19 pandemic that has developed over recent months. It is difficult to find events in history that have affected all humanity in such a global way and that, at the same time, have revealed so clearly the healthcare disparities existing in our societies. Obvious examples are the overwhelming mortality differences between different social and ethnic groups, as evidenced by the increased mortality rate among African Americans and Hispanics in the US [14].

Since the beginning of the health emergency, there have been several calls from the social scientific community to try to understand how people cope with stress and make health decisions related to the recommendations of health authorities in the new context [15]. We could say that the current pandemic has made contextualization mainstream. But what does the pandemic really imply in terms of psychosocial well-being? The intensification of the exclusion conditions of the less favored, extreme pressure on health and supply services workers, a lack of perspective for young generations, the proliferation of conspiracy theories that could hinder the implementation of solutions, etc. It is clearly not the first time that these circumstances have happened, some are typical of more localized emergencies such as natural disasters, so perhaps the novelty lies in that it has happened at almost the same time for all humanity.

It seems obvious to say that this context switch has made it necessary to adapt many lines of research to be useful in solving problems in the new reality. However, the debate on the need for the contextualization of psychological research has long been fraught with controversy. The debate has always been between those who believe that theories and their applications should be anchored in history, ideologies, and social power relations and those who rather opt for universalistic applications. The latter, in our opinion dominant in the areas of mental health and well-being research, can be perceived as pragmatic or, on the contrary, decontextualized by the former. The reality is that the promotion of psychosocial well-being with formulas that do not sufficiently consider the state of the recipients in terms of their status of social power (i.e., if they are object of stigmatization and exclusion or on the contrary, enjoy a privileged status compared to other groups), is common. These are uncomfortable topics, which are not usually covered in depth in handbooks dedicated to the conceptual understanding or the application of techniques to increase well-being.

Although not all the articles included in this Special Issue have engaged with the epistemological depth that the aforementioned discussions might require, we are happy to have provoked a debate on the need to consider the context of complex and multidimensional problems when presenting research results on psychosocial well-being.

## 2. The Works Included

Table 1 shows a synthesis of all articles accepted for publication in the Special Issue “Understanding Psychosocial Well-being in the Context of Complex and Multidimensional Problems”. The submission of articles was open from October 2018 to December 2019. We received a total of 42 submissions, of which 11 were finally accepted. The methods of the accepted publications include mostly cross-sectional studies, although psychometric instrument validations, a randomized controlled trial and a qualitative study were also included. Populations are varied, ranging from the general population or university students, to specific populations such as Huntington’s disease caregivers or an online suicidal community.

Freire and colleagues [16] performed mediation and moderation models to test the role of self-efficacy in the relationship between eudaimonic well-being and the use of adaptive coping strategies in the academic context. Their results showed that self-efficacy partially mediates but does not moderate the relationship between eudaimonic well-being and adaptive coping strategies. Therefore, self-efficacy constitutes a relevant personal resource that favors adequate coping with academic stress, but it is insufficient by itself when the level of eudaimonic well-being of students is low.

Wang and colleagues [17] labeled a total of 4489 postings (from a total of 560,000 analyzed) of people with suicidal ideation in a Chinese online suicidal community. Their results revealed that people with suicidal ideation are significantly more active than other users in the community. Using social network analysis, they also found that the more frequently users communicate with people with suicidal ideation, the more likely it was that they would become suicidal. In addition, Chinese women may be more likely to be at risk of suicide than men in the community. Their study stresses the importance of suicide prevention in suicidal communities on social media, especially for those individuals who might not seek mental health services.

Eiroa-Orosa and Limiñana-Bravo [18] carried out the validation of an instrument to measure mental health professionals’ beliefs and attitudes towards service users’ rights. Using exploratory and confirmatory factor analyses, as well as item response theory methods within a sample of 480 questionnaires filled out by mental health professionals, they found a four-factor solution consisting of system criticism/justifying beliefs, freedom/coercion, empowerment/paternalism, and tolerance/discrimination. The scale can be used to measure the impact of recovery and anti-stigma “targeted, local, credible, continuous contact” methodology-based interventions carried out with mental health professionals.

Lee et al. [19] evaluated the efficacy of short-term internet-delivered cognitive–behavioral therapy (ICBT) on female nursing practicum students with irritable bowel syndrome. They performed a cluster randomized controlled trial comprising 160 participants, which were divided into three groups: (1) ICBT, (2) expressive writing (EW), and (3) wait-list control. Levels of anxiety, depression, and irritable bowel syndrome symptoms were assessed at four time points: baseline assessment at T0, 2 weeks after T0 (T1), at the end of practicum (T2), and at a 3-month follow-up (T3). The results showed that the ICBT and EW groups exhibited a significant, yet small, reduction in anxiety and depression at T2 and T3 compared to the wait-list control group. The EW group exhibited a significantly greater reduction in anxiety and depression compared to the ICBT group at T2. However, the ICBT group demonstrated greater improvements in alleviating anxiety and depression at T3 compared to the EW group. Therefore, EW and ICBT show similar effects in the short term, but ICBT performs better over the longer term.

Kim et al. [20] enrolled 685 firefighters in their study on the work limitations derived by the exposure to work-related traumatic events. A strong relationship between the firefighters’ exposure to work-related traumatic events and their work limitations was demonstrated, whether they were physical, psychosocial, or environmental. The authors stress the need for a professional care management system for firefighters to prevent and manage work-related traumatic events, protecting and improving their performance.

Arias-de la Torre et al. [21] analyzed data on the psychological distress of 4166 first-year university students from nine universities across Spain. Gender-stratified analyses showed that 46.9% of men and 54.2% of women had psychological distress. Psychological distress levels increased as family support decreased for both genders. However, an association between psychological distress with employment status was found only for women. Both family support and the student’s employment status could be particularly relevant factors in developing prevention strategies against the onset of mental health diseases in this specific population.

Chen et al. [22] analyzed the potential psychological mechanisms of subjective well-being in migrant workers using structural equation models. The investigators randomly recruited 2573 migrant workers from Shanghai. Subjective well-being was predicted by distress, sense of coherence and generalized resistant resources (income ratio, education attainment, marital status, family accompaniment, preventive activity). The underlying mechanisms unveiled by the model included a mediating role of the sense of coherence between adverse dilemmas and well-being and a mediation of generalized resistant resources on the relationship between the sense of coherence and well-being. Based on the results of the study, approaches such as fostering social bonds and developing activities aimed at promoting marital relations and social welfare could be particularly promising for improving migrant worker well-being.

Bartoszek et al. [23] explored the reliability and validity of the Huntington’s disease quality of life battery for caretakers within a Polish population. Data from 90 caretakers were subjected to principal component and reliability analyses. The Polish version of the instrument demonstrated good internal consistency and congruent validity. The authors conclude that the Polish version of the shortened version of the questionnaire is similarly valid compared to the original English version and is suitable for use within this population.

Codina and Pestana [24] analyzed the time spent on the leisure activities, leisure experience (i.e., perceptions of freedom and satisfaction) and the five factors of time perspective (hedonistic and fatalistic present; positive and negative past; and future orientation) of 869 participants (435 men and 434 women). Their results show significant gender differences. Men had more leisure time, but women had a more positive leisure experience and time perspective than men. The authors concluded that women enjoy themselves more with less available leisure time and are more positive regarding time orientations.

Moriyama et al. [25] described the subjective well-being of older residents in Fukushima Prefecture seven years and seven months after the Great East Japan Earthquake and examined the effect of relocation to restoration public housing on subjective well-being, social capital, and health indicators. They also investigated the association between social capital and subjective well-being. Older restoration public housing residents may demonstrate lower social capital and health indicators after the earthquake. Mistrust was found to be positively associated with low subjective well-being in these residents. The authors propose that future studies should examine the relationship between subjective well-being and social capital, as well as the effectiveness of support for enhancing the trust of older restoration public housing residents regarding the involvement of scientists and practitioners in promoting subjective well-being.

Rodríguez-Cifuentes et al. [26] tested the mediating role of psychological capital between motivational orientations and their organizational consequences. Spanish employees aged over 40 (n = 741), were recruited in two waves with a 4-month interval. Their results support the hypothesis that psychological capital resources may play a mediating role in the relationship between motivational traits and performance in the form of extra-role behaviors (both positive and negative for the company) and that approach orientation traits are mainly related to a better performance, fostering organizational citizenship behaviors and diminishing counterproductive work behavior. The findings show that employees who develop their personal resources may have a positive impact on their organizations. One way of developing employee motivation at a cognitive level would be to understand situations more positively, reinforcing ideas of approach over those of avoidance. A clear implication of the study is that every employee should be treated according to their personal needs and that one-size-fits-all approaches are not effective.

## 3. Conclusions

Human beings develop in complex environments where the determinants of psychosocial well-being are multivariate. These relationships might depend on power imbalances caused by gender, economic, ethnic or ability inequalities. Researchers interested in understanding psychosocial well-being must consider the contexts they study and the complexity of the multiple interactions that can occur in them. Although there may be universal relationships, it is important not to take this for granted when conducting research outside mainstream study populations. What works for a wealthy white Caucasian male in terms of self-efficacy, locus of control, sense of coherence, etc., might not be the same for people of other cultures, with different worldviews and/or are subjected to any kind of injustice. Last, but not least, the beliefs and attitudes of researchers and practitioners should be both an object of study and an element of reflection. No one is free from prejudice, even if using supposedly “objective” methods. Awareness of and reflection on the possible biases of our research and practice methods are the only way to reduce bad practices caused by prejudices.

## Figures and Tables

**Table 1:** Description of the articles included in the Special Issue in chronological publishing order.

Authorship	Research Context	Location	Methodology	Outcomes	Analyses	Main Findings
Freire et al. [16]	University students	A Coruña, Spain	Quantitative, cross-sectional	Eudaimonic well-being, self-efficacy, adaptive coping strategies	Mediation	Self-efficacy partially mediates but does not moderate the relationship between eudaimonic well-being and adaptive coping strategies
Wang et al. [17]	Suicidal online community	China (online)	Content analysis	Suicidal ideation, methods, thanatophobia, attempts, search for partners	Social network analysis	The more frequently users communicate with people with suicidal ideation, the more likely users become suicidal
Eiroa-Orosa and Limiñana-Bravo [18]	Mental health professionals	Spain	Psychometric instrument validation	Mental health professionals’ beliefs and attitudes towards service users’ rights	Exploratory and confirmatory factor and item response theory analyses	Four-factor solution consisting of system criticism/justifying beliefs, freedom/coercion, empowerment/paternalism, and tolerance/discrimination
Lee et al. [19]	Nursing students with irritable bowel syndrome (IBS)	Taiwan	Cluster randomized controlled trial comparing internet delivered cognitive–behavioral therapy (ICBT), expressive writing and a waitlist	Bowel symptom severity, anxiety, depression	ANCOVA	IBS symptoms, depression, and anxiety decreased over time for all three tested groups with one exception: the ICBT group’s IBS scores increased slightly at the end of treatment
Kim et al. [20]	Firefighters	Korea	Quantitative, cross-sectional	Traumatic event experience, depression, quality of life, health-related work limitations	Multivariable logistic regression analysis	A relationship between the firefighters’ exposure to work-related traumatic events and their work limitations was found
Arias-de la Torre et al. [21]	First-year university students	Spain	Quantitative, cross-sectional	Psychological distress	Multivariate logistic regression models	In both genders, psychological distress levels increased as family support decreased. Among women, psychological distress was associated with their employment status
Chen et al. [22]	Migrant workers	Shanghai, China	Quantitative, cross-sectional	Distress, well-being, sense of coherence (SOC)	Multiple linear regression and structural equation models	Migrant workers with low SOC and high distress are vulnerable to poor well-being levels. Meanwhile, income and marital status may strengthen SOC
Bartoszek et al. [23]	Huntington’s disease caregivers	Poland	Psychometric instrument cultural validation	Huntington’s disease caregivers’ quality of life (HDQoL-C)	Principal component analysis and Cronbach’s α coefficients	The Polish version of the shortened version of the HDQoL-C is similarly valid compared to the original English version
Codina and Pestana [24]	General population	Spain	Quantitative, cross-sectional	Leisure experience, time perspective	Bivariate analyses	The results show significant gender differences: men have more leisure time, but women have a more positive leisure experience and time perspectives than men
Moriyama et al. [25]	Older adults relocated to restoration public housing	Fukushima, Japan	Quantitative, cross-sectional	Subjective well-being	Bivariate and multivariate (multiple regression) analyses	Older restoration public housing residents may demonstrate lower social capital and health indicators after the Great East Japan Earthquake. Mistrust was found to be positively associated with low subjective well-being in restoration public housing residents
Rodríguez-Cifuentes et al. [26]	Employees	Spain	Quantitative, cross-sectional	Motivational traits, psychological capital, work performance	Mediation	Psychological capital resources may play a mediating role and approach orientation traits are mainly related to a better performance, fostering organizational citizenship behaviors, and diminishing counterproductive work behavior.
